# Dataset for file fragment classification of textual file formats

**DOI:** 10.1186/s13104-019-4837-4

**Published:** 2019-12-11

**Authors:** Fatemeh Mansouri Hanis, Mehdi Teimouri

**Affiliations:** Information Theory and Coding Laboratory, University of Tehran, Tehran, Iran

**Keywords:** Classification, File formats, File fragments, Textual file formats

## Abstract

**Objectives:**

Classification of textual file formats is a topic of interest in network forensics. There are a few publicly available datasets of files with textual formats. Therewith, there is no public dataset for file fragments of textual file formats. So, a big research challenge in file fragment classification of textual file formats is to compare the performance of the developed methods over the same datasets.

**Data description:**

In this study, we present a dataset that contains file fragments of five textual file formats: Binary file format for Word 97–Word 2003, Microsoft Word open XML format, portable document format, rich text file, and standard text document. This dataset contains the file fragments in three different languages: English, Persian, and Chinese. For each pair of file format and language, 1500 file fragments are provided. So, the dataset of file fragments contains 22,500 file fragments.

## Objective

A considerable amount of Internet traffic is used for exchanging file formats that merely carry textual data. As the sizes of these files are usually much bigger than the maximum network packet size, the files are segmented into fragments. The fragments generated by various users are transmitted over the network. Some of these fragments can be received by the network surveillance unit. The network surveillance unit may wish to detect the file format of each fragment for network forensics purposes.

Many researches have been carried in the field of file fragment classification of textual file formats [1–6]. There are a few publicly available datasets of files with different formats [7]. Therewith, there is no public dataset for file fragments of textual file formats. So, most of the mentioned researches exclusively use their own private datasets. This makes it difficult for other researchers to compare the proposed methods with the existing methods.

In this study, we present a dataset that contains file fragments of five textual file formats: Binary file format for Word 97–Word 2003 (DOC), Microsoft Word open XML format (DOCX), portable document format (PDF), rich text file (RTF), and standard text document (TXT). This dataset includes the file fragments in three different languages: English (EN), Persian (FA), and Chinese (CH).

## Data description

First, the whole set of textual files are gathered. These files are in three different languages: English, Persian, and Chinese. The English textual files are in four different formats: DOC, DOCX, TXT, and RTF. These files are gathered from the freely available forensic research data collected by Garfinkel et al. [8]. We have converted a subset of English DOC files to obtain the set of English PDF files. So, we have textual files in five formats: DOC, DOCX, TXT, PDF, and RTF.

For the Persian and Chinese languages, we have searched for DOC files in google.com with many different keywords and phrases. Then, we have converted different subsets of these DOC files into the other four formats: DOCX, RTF, TXT, and PDF. TXT files in all three languages are saved in Universal Transformation Format-8 (UTF-8) format. It should be noted that regardless of file format, the content of any pair of files is not the same. In other words, when we convert a file from a specific format to another format, the original file is removed from the set of files.

For each pair of file format and language, we have collected 300 different files. So, totally we have 4500 files. Each of these files is segmented into 1 Kbyte (i.e. 1024 bytes) fragments. Then, five fragments are randomly selected among the fragments of each file. Before randomly selecting the fragments, 12.5% of the initial fragments and 12.5% of the final fragments of each file are discarded. This is to ensure that the fragments do not contain the file headers or trailers.

For each pair of file format and language, we have 1500 file fragments. So, the dataset of file fragments contains 22,500 file fragments. The dataset is partitioned according to 15 different pairs of file format and language. Each partition is represented by an individual data file shown in Table 1. For example, data file 6 (i.e. DOC-FA.dat) contains 1500 fragments of DOC files in the Persian language. Data files are provided in a generic binary data file format with .dat file extension. Data file 16 (i.e. ReadFragments.m) is a script in MATLAB language that reads all the fragments from a specific data file. This script is written specifically to accompany this dataset. By running this script and selecting a data file, the fragments contained in this dataset are read and stored in a variable name Dataset. Variable Dataset is a MATLAB structure array with only one field named fragments. Dataset(j).fragments (j = 1,2,…,300) is a cell array with length 5 that contains five fragments of the jth file in the selected data file.

**Table 1:** Overview of data files/data files

Label	Name of data file/data file	File types (file extension)	Data repository (DOI)
Data file 1	DOC-EN	Generic binary data (.dat)	OSF (10.17605/OSF.IO/4N8RT)
Data file 2	DOCX-EN	Generic binary data (.dat)	OSF (10.17605/OSF.IO/4N8RT)
Data file 3	PDF-EN	Generic binary data (.dat)	OSF (10.17605/OSF.IO/4N8RT)
Data file 4	TXT-EN	Generic binary data (.dat)	OSF (10.17605/OSF.IO/4N8RT)
Data file 5	RTF-EN	Generic binary data (.dat)	OSF (10.17605/OSF.IO/4N8RT)
Data file 6	DOC-FA	Generic binary data (.dat)	OSF (10.17605/OSF.IO/4N8RT)
Data file 7	DOCX-FA	Generic binary data (.dat)	OSF (10.17605/OSF.IO/4N8RT)
Data file 8	PDF-FA	Generic binary data (.dat)	OSF (10.17605/OSF.IO/4N8RT)
Data file 9	TXT-FA	Generic binary data (.dat)	OSF (10.17605/OSF.IO/4N8RT)
Data file 10	RTF-FA	Generic binary data (.dat)	OSF (10.17605/OSF.IO/4N8RT)
Data file 11	DOC-CH	Generic binary data (.dat)	OSF (10.17605/OSF.IO/4N8RT)
Data file 12	DOCX-CH	Generic binary data (.dat)	OSF (10.17605/OSF.IO/4N8RT)
Data file 13	PDF-CH	Generic binary data (.dat)	OSF (10.17605/OSF.IO/4N8RT)
Data file 14	TXT-CH	Generic binary data (.dat)	OSF (10.17605/OSF.IO/4N8RT)
Data file 15	RTF-CH	Generic binary data (.dat)	OSF (10.17605/OSF.IO/4N8RT)
Data file 16	ReadFragments	Matlab script file (.m)	OSF (10.17605/OSF.IO/4N8RT)

## Limitations

- There are other formats of textual files such as Hypertext Markup Language (HTML) format and Cascade Styling Sheets (CSS) format that are not included in the dataset.
- Multi-language documents and documents in other commonly used languages such as German, Italian, Spanish, and French are not considered for the construction of the dataset.
- The size of the fragments is considered to be fixed and equal to 1024 bytes.

## Data Availability

The data described in this Data note can be freely and openly accessed on OSF at 10.17605/OSF.IO/4N8RT [9]. Please see Table 1 and reference list for details and links to the data.

## Abbreviations

CH: Chinese language class
DOC: binary file format for Word 97–Word 2003
DOCX: Microsoft Word open XML format
EN: English language class
FA: Persian language class
HTML: hypertext markup language
PDF: portable document format
RTF: rich text file
TXT: standard text document
UTF-8: Universal Transformation Format-8
